# Mosaic nanoparticles elicit cross-reactive immune responses to zoonotic coronaviruses in mice

**DOI:** 10.1126/science.abf6840

**Published:** 2021-01-12

**Authors:** Alexander A. Cohen, Priyanthi N. P. Gnanapragasam, Yu E. Lee, Pauline R. Hoffman, Susan Ou, Leesa M. Kakutani, Jennifer R. Keeffe, Hung-Jen Wu, Mark Howarth, Anthony P. West, Christopher O. Barnes, Michel C. Nussenzweig, Pamela J. Bjorkman

**Affiliations:** 1 Division of Biology and Biological Engineering, California Institute of Technology, Pasadena, CA 91125, USA.; 2 Department of Biochemistry, University of Oxford, Oxford OX1 3QU, UK.; 3 Laboratory of Molecular Immunology, The Rockefeller University, New York, NY 10065, USA.

## Abstract

Protection against severe acute respiratory syndrome coronavirus 2 (SARS-CoV-2) and SARS-related emergent zoonotic coronaviruses is urgently needed. We made homotypic nanoparticles displaying the receptor binding domain (RBD) of SARS-CoV-2 or co-displaying SARS-CoV-2 RBD along with RBDs from animal betacoronaviruses that represent threats to humans (mosaic nanoparticles with four to eight distinct RBDs). Mice immunized with RBD nanoparticles, but not soluble antigen, elicited cross-reactive binding and neutralization responses. Mosaic RBD nanoparticles elicited antibodies with superior cross-reactive recognition of heterologous RBDs relative to sera from immunizations with homotypic SARS-CoV-2–RBD nanoparticles or COVID-19 convalescent human plasmas. Moreover, after priming, sera from mosaic RBD–immunized mice neutralized heterologous pseudotyped coronaviruses as well as or better than sera from homotypic SARS-CoV-2–RBD nanoparticle immunizations, demonstrating no loss of immunogenicity against particular RBDs resulting from co-display. A single immunization with mosaic RBD nanoparticles provides a potential strategy to simultaneously protect against SARS-CoV-2 and emerging zoonotic coronaviruses.

## Fighting zoonotic coronaviruses

In the past 20 years, three betacoronaviruses thought to have originated in bats have caused devastating disease in humans. The global pandemic caused by the latest such virus, severe acute respiratory syndrome coronavirus 2 (SARS-CoV-2), highlights the need to protect against other strains that could present a threat to humans. Cohen *et al.* constructed nanoparticles displaying the protein domain that binds the host cell receptor (receptor-binding domain or RBD), either a homotypic SARS-CoV-2 particle or mosaic particles displaying RBDs from four or eight different betacoronaviruses. In mice, antibodies to the SARS-CoV-2 RBD were elicited just as well by mosaic particles as by homotypic nanoparticles. The mosaic nanoparticles elicited antibodies that, beyond recognizing the strains displayed, also recognized mismatched strains.

*Science*, this issue p. 735

## Teaser

Immunizing with nanoparticles that display diverse coronavirus receptor binding domains elicits cross-reactive and neutralizing antibody responses.

Severe acute respiratory syndrome coronavirus 2 (SARS-CoV-2), a newly emergent betacoronavirus, resulted in a global pandemic in 2020, infecting millions and causing the respiratory disease COVID-19 (*1*, *2*). Two other zoonotic betacoronaviruses, SARS-CoV and Middle East respiratory syndrome coronavirus (MERS-CoV), have also resulted in outbreaks within the past 20 years (*3*). All three viruses presumably originated in bats (*4*), with SARS-CoV and MERS-CoV adapting to intermediary animal hosts before jumping to humans. SARS-like viruses circulate in bats, and serological surveillance of people living near caves where bats carry diverse coronaviruses demonstrates direct transmission of SARS-like viruses with pandemic potential (*5*). This finding suggests that a pan-coronavirus vaccine is needed to protect against future outbreaks and pandemics. In particular, the bat WIV1 and SHC014 strains are thought to represent an ongoing threat to humans (*6*, *7*).

**Fig. 1:**
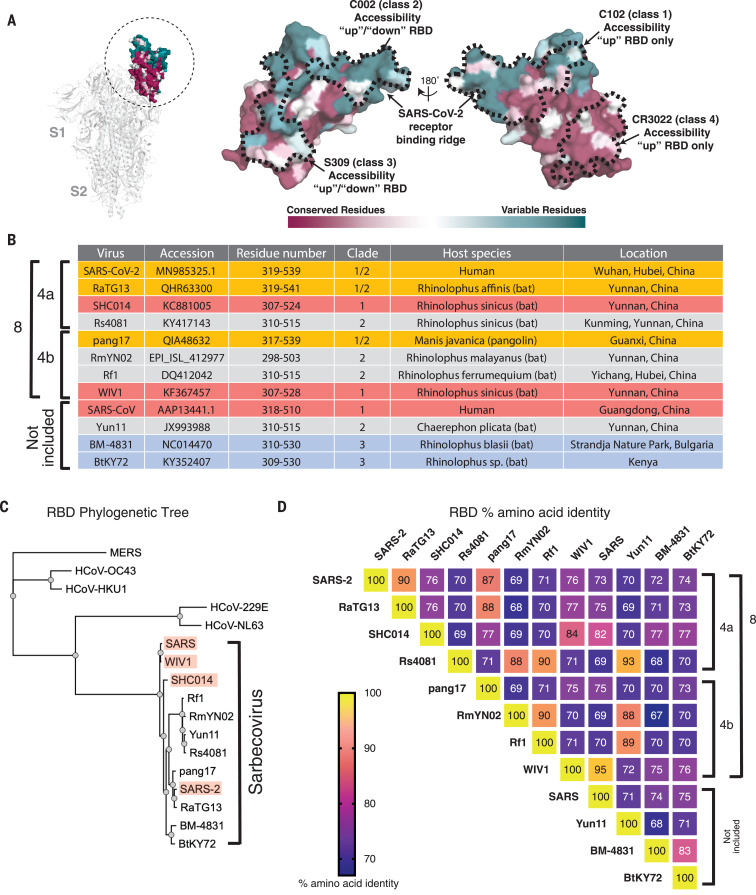
Properties of RBDs chosen for this study. (**A**) Left: Structure of SARS-CoV-2 S trimer (PDB 6VXX) with one RBD (dashed circle) in an “up” position. Center and right: Sequence conservation of 12 RBDs calculated by the ConSurf Database (*49*) plotted on a surface representation of the RBD structure (PDB 7BZ5). Epitopes for representatives from defined classes of RBD-binding antibodies (classes 1 to 4) (*24*) are indicated by dashed lines. (**B**) Summary of properties of the viral strains from which the 12 sarbecovirus RBDs were derived. (**C**) Phylogenetic tree of human and selected other coronaviruses based on RBD protein sequences. Red shading indicates strains known to use ACE2 as a receptor. (**D**) Heat map showing percent amino acid sequence identities among 12 sarbecovirus RBDs.

Most current SARS-CoV-2 vaccine candidates include the spike trimer (S), the viral protein that mediates target cell entry after one or more of its receptor binding domains (RBDs) adopts an “up” position to bind a host receptor (Fig. 1A). The RBDs of human coronaviruses SARS-CoV-2, SARS-CoV, and HCoV-NL63, as well as those of the related animal coronaviruses WIV1 and SHC014, use angiotensin-converting enzyme 2 (ACE2) as their host receptor (*1*, *8*, *9*), whereas other coronaviruses use receptors such as dipeptidyl peptidase 4 (*10*) or sialic acids (*11*, *12*). Consistent with its function in viral entry, S is the primary target of neutralizing antibodies (*13*–*22*), with many targeting the RBD (*14*–*18*, *21*–*26*).

Multivalent display of antigen enhances B cell responses and can provide longer-lasting immunity than monovalent antigens (*27*, *28*); thus, protein-based vaccine candidates often involve a nanoparticle that enables antigen multimerization. Many nanoparticles and coupling strategies have been explored for vaccine design (*29*), with “plug and display” strategies being especially useful (*30*, *31*). In one such approach, multiple copies of an engineered protein domain called SpyCatcher fused to subunits of a virus-like particle form spontaneous isopeptide bonds to purified antigens tagged with a 13-residue SpyTag (*29*–*32*). The SpyCatcher-SpyTag system was used to prepare multimerized SARS-CoV-2 RBD or S trimer that elicited high titers of neutralizing antibodies (*33*, *34*). Although promising for protection against SARS-CoV-2, coronavirus reservoirs in bats suggest future cross-species transmission (*6*, *7*, *35*), necessitating a vaccine that protects against emerging coronaviruses as well as SARS-CoV-2. Here, we prepared SpyCatcher003-mi3 nanoparticles (*31*, *36*) simultaneously displaying SpyTagged RBDs from human and animal coronaviruses to evaluate whether mosaic particles can elicit cross-reactive antibody responses, as previously demonstrated for influenza head domain mosaic particles (*37*). We show that mice immunized with homotypic or mosaic nanoparticles produced broad binding and neutralizing responses, in contrast to plasma antibodies elicited in humans by SARS-CoV-2 infection. Moreover, relative to homotypic SARS-CoV-2 nanoparticles, mosaic nanoparticles showed enhanced heterologous binding and neutralization properties against human and bat SARS-like betacoronaviruses (sarbecoviruses).

We used a study of sarbecovirus RBD receptor usage and cell tropism (*38*) to guide our choice of RBDs for co-display on mosaic particles. From 29 RBDs that were classified into distinct clades (clades 1, 2, 1/2, and 3) (*38*), we identified diverse RBDs from SARS-CoV, WIV1, and SHC014 (clade 1); SARS-CoV-2 (clade 1/2); Rs4081, Yunnan 2011 (Yun11), and Rf1 (clade 2); and BM-4831 (clade 3). Of these, SARS-CoV-2 and SARS-CoV are human coronaviruses and the rest are bat viruses originating in China or Bulgaria (BM-4831). We also included RBDs from the GX pangolin clade 1/2 coronavirus (referred to here as pang17) (*39*); RaTG13, the bat clade 1/2 virus most closely related to SARS-CoV-2 (*40*); RmYN02, a clade 2 bat virus from China (*41*); and BtKY72, a Kenyan bat clade 3 virus (*42*). Mapping of the sequence conservation across selected RBDs showed varying degrees of sequence identity (68 to 95%), with highest sequence variability in residues corresponding to the SARS-CoV-2 ACE2 receptor binding motif (Fig. 1, A to D, and fig. S1). We chose 8 of the 12 RBDs as sources for three types of mosaic nanoparticles—mosaic-4a (coupled to SARS-2, RaTG13, SHC014, and Rs4081 RBDs); mosaic-4b (coupled to pang17, RmYN02, Rf1, and WIV1 RBDs); and mosaic-8 (coupled to all eight RBDs)—and compared them with homotypic mi3 particles constructed from SARS-CoV-2 RBD alone (homotypic SARS-2). RBDs from SARS, Yun11, BM-4831, and BtKY72, which were not coupled to mosaic particles, were used to evaluate sera for cross-reactive responses.

**Fig. 2:**
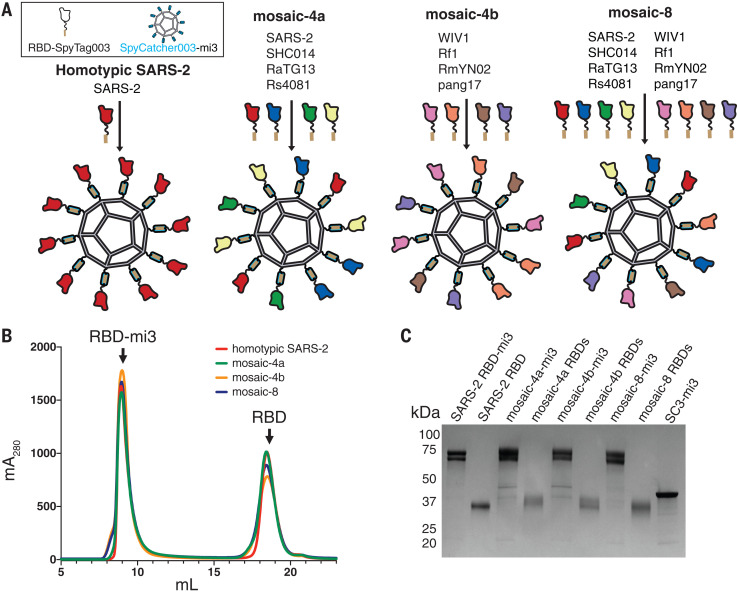
Construction of RBD nanoparticles. (**A**) Left: SpyTagged RBDs were attached to SpyCatcher003-mi3 to make a homotypic particle and three mosaic particles. There are 60 potential coupling sites on mi3; only 10 are shown for clarity. (**B**) SEC profile showing separation of RBD nanoparticles and free RBD proteins. (**C**) Coomassie-stained SDS-PAGE of RBD-coupled nanoparticles, free RBD proteins, and uncoupled SpyCatcher003-mi3 particles (SC3-mi3).

SpyTag003-RBDs were coupled to SpyCatcher003-mi3 (60 potential conjugation sites) (*36*, *43*) to make homotypic and mosaic nanoparticles (Fig. 2A). Particles were purified by size exclusion chromatography (SEC) and analyzed by SDS–polyacrylamide gel electrophoresis (PAGE), revealing monodisperse SEC profiles and nearly 100% conjugation (Fig. 2, B and C). Representative RBDs were conjugated to SpyCatcher003-mi3 with similar or identical efficiencies (fig. S2), which suggests that mosaic particles contained approximately equimolar mixtures of different RBDs.

**Fig. 3:**
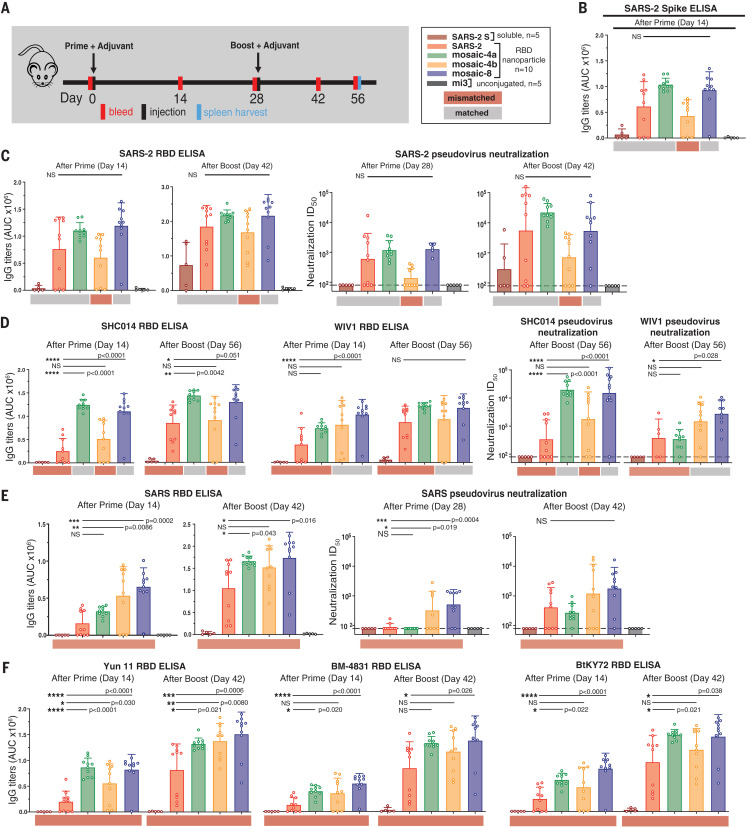
RBD nanoparticles induce cross-reactive IgG responses in immunized mice. Red and gray rectangles below ELISA and neutralization data represent mismatched strains (red; RBD from that strain was not present on the immunized particle) or matched strains (gray; RBD was present on the immunized particle). (**A**) Left: Immunization schedule; adjuvant is AddaVax (Invivogen). Right: Key for immunizations; number of mice in each cohort is indicated. (**B** to **F**) Neutralization and/or binding data for serum IgGs for recognition of (B) SARS-2 spike trimer, (C) SARS-2 RBD and SARS-2 pseudovirus, (D) SHC014 and WIV1 RBDs and corresponding pseudoviruses, (E) SARS RBD and SARS pseudovirus, and (F) Yun11, BM-4831, and BtKY72 RBDs. Mice were immunized with soluble SARS-CoV-2 S trimer (SARS-2 S; brown bars) or the following nanoparticles: homotypic SARS-2 (red), mosaic-4a (green), mosaic-4b (yellow), mosaic-8 (blue), or unconjugated SpyCatcher003-mi3 (mi3; black). ELISA data from serum IgG responses to SARS-2 spike trimer (B) or RBDs [(C) to (F)] are shown as area under the curve (AUC). For (C) to (E), neutralization potencies are presented as half-maximal inhibitory dilutions (ID_50_ values) of sera against the pseudoviruses from the indicated coronavirus strains. Dashed horizontal lines correspond to the lowest dilution representing the limit of detection. Each dot represents serum from one animal, with means and SDs for vaccinated cohorts denoted by rectangles and horizontal lines, respectively. Significant differences between groups linked by horizontal lines are indicated by asterisks and *P* values. NS, not significant.

We immunized mice with soluble SARS-CoV-2 spike trimer (SARS-2 S), nanoparticles displaying only SARS-2 RBD (homotypic SARS-2), nanoparticles co-displaying RBDs (mosaic-4a, mosaic-4b, or mosaic-8), or unconjugated nanoparticles (mi3). Immunoglobulin G (IgG) responses were evaluated after prime or boost immunizations (Fig. 3A) by enzyme-linked immunosorbent assay (ELISA) against SARS-2 S (Fig. 3B) or a panel of RBDs (Fig. 3, C to F, and fig. S3). Sera from unconjugated nanoparticle-immunized animals (Fig. 3 and fig. S3, black) showed no responses above background. Anti–SARS-2 S trimer and anti–SARS-2 RBD serum responses were similar (Fig. 3, B and C), demonstrating that antibodies elicited against RBDs can access their epitopes on SARS-2 S trimer. We also conducted in vitro neutralization assays using a pseudotyped virus assay that quantitatively correlates with authentic virus neutralization (*44*) for strains known to infect 293T_ACE2_ target cells (SARS-CoV-2, SARS, WIV1, and SHC104). Neutralization and ELISA titers were significantly correlated (fig. S4), which implies that ELISAs are predictive of neutralization results when viral entry receptor usage prevents accurate pseudotyped neutralization assays.

Mice immunized with soluble SARS-2 S trimer showed no binding or neutralization except for autologous responses against SARS-2 after boosting (Fig. 3, C to F, brown bars). By contrast, sera from RBD nanoparticle–immunized animals exhibited binding to all RBDs (Fig. 3, C to F, red, green, yellow, and blue bars; fig. S3A) and neutralization against all four strains after boosting (Fig. 3, C to E), consistent with increased immunogenicities of multimerized antigen on nanoparticles versus soluble antigen (*27*, *28*). Homotypic SARS-2 nanoparticles, but not soluble SARS-2 trimer, induced heterologous responses to zoonotic RBDs and neutralization of heterologous coronaviruses (Fig. 3, D to F). To address whether co-display of SARS-2 RBD along with other RBDs on mosaic-4a and mosaic-8 versus homotypic display of SARS-2 RBD (homotypic SARS-2) diminished anti–SARS-2 responses, we compared SARS-2–specific ELISA and neutralization titers for mosaic versus homotypic immunizations (Fig. 3C); there were no significant differences in IgG anti–SARS-2 titers for animals immunized with homotypic (Fig. 3C, red) versus mosaic nanoparticles (Fig. 3C, green and blue). Thus, in terms of the magnitude of immune response against SARS-2, there was no advantage of immunization with a homotypic RBD nanoparticle versus a mosaic nanoparticle that included SARS-2 RBD.

We next compared serum responses against matched RBDs (RBDs present on an injected nanoparticle) versus mismatched RBDs (RBDs not present on an injected nanoparticle) (Fig. 3 and fig. S3, gray and red horizontal shading, respectively). Although SARS-2 RBD was not presented on mosaic-4b, antibody titers elicited by mosaic-4b immunization (yellow) were not significantly different from titers elicited by matched nanoparticle immunizations [homotypic SARS-2 (red), mosaic-4a (green), and mosaic-8 (blue)], and sera from boosted mosaic-4b–immunized mice neutralized SARS-2 pseudovirus (Fig. 3C). In other matched versus mismatched comparisons, sera showed binding and neutralization of SHC014 and WIV1 regardless of whether these RBDs were included on the injected nanoparticle (Fig. 3D); this result implies sharing of common epitopes among RBDs (Fig. 1A).

In an experiment that demonstrated the advantages of mosaic versus homotypic SARS-2 nanoparticles, sera from mosaic-8–immunized mice bound SHC014 and WIV1 RBDs significantly better after priming than sera from homotypic SARS-2–immunized mice and retained better binding to SHC014 RBD after boosting (Fig. 3D). Thus, the potential increased avidity of the homotypic SARS-2 nanoparticle displaying only one type of RBD over the mosaic-8 nanoparticles did not confer increased breadth. Moreover, mosaic-8–immunized and boosted sera were more potent than sera from homotypic SARS-2–immunized animals in neutralizing SHC014 and WIV1 (Fig. 3D). Neutralization of the SHC014 and WIV1 pseudoviruses by mosaic-8 sera suggests that combining RBDs on a mosaic nanoparticle does not diminish the immune response against a particular RBD, as also suggested by ELISA binding of sera to Rs4081 and RaTG13 (fig. S3, A and B).

To further address whether RBD nanoparticles elicited antibodies that recognized totally mismatched strains and SARS-CoV-2 RBD mutants, we evaluated sera for binding to SARS, Yun11, BM-4831, and BtKY72 RBDs (Fig. 3, E and F); SARS-2 RBD mutants (fig. S3C); and MERS-CoV RBD (fig. S3D), as well as for neutralization in SARS pseudovirus assays (Fig. 3E). We found no reductions in SARS-2 RBD binding as a result of mutations [Y453F, the “Danish mink variant” (*45*), or a Q493K/Q498Y/P499T triple mutant (*46*)] (fig. S3C), no binding of any elicited sera to MERS-CoV RBD (fig. S3D), and higher and more cross-reactive antibody responses for mosaic immunizations than for homotypic SARS-2 immunizations (e.g., mosaic-8–primed and boosted animals showed significantly higher titers against SARS RBD than did sera from homotypic SARS-2–immunized mice) (Fig. 3E). After priming, sera from the homotypic SARS-2–immunized animals did not neutralize SARS, whereas the mosaic-4b and mosaic-8 sera were neutralizing (Fig. 3E), perhaps because these nanoparticles included WIV1 RBD, which is related by 95% amino acid identity to SARS RBD (Fig. 1D). After boosting, SARS-2 and mosaic-4a sera were also neutralizing, although titers were lower than for mosaic-8–immunized animals by a factor of ~4 (Fig. 3E). ELISA titers against other mismatched RBDs (Yun11, BM-4831, and BtKY72) were significantly higher for sera collected after mosaic-8 priming than for sera from homotypic SARS-2 priming, and heightened binding was retained after boosting (Fig. 3F). Thus, relative to homotypic SARS-2 nanoparticles, mosaic nanoparticles (particularly mosaic-8) induce higher antibody titers against mismatched RBDs, This is another finding that favors the co-display approach for inducing broader anti-coronavirus responses, especially after a single prime.

Using flow cytometry, we investigated the potential for cross-reactive recognition—specifically, whether B cell receptors on IgG^+^ splenic B cells from RBD nanoparticle–boosted animals could simultaneously recognize RBDs from SARS-2 and Rs4081 (related by 70% sequence identity) (Fig. 1D and fig. S5). Whereas control animals were negative, all other groups showed B cells that recognized SARS-2 and Rs4081 RBDs simultaneously, suggesting the existence of antibodies that cross-react with both RBDs (fig. S5E).

**Fig. 4:**
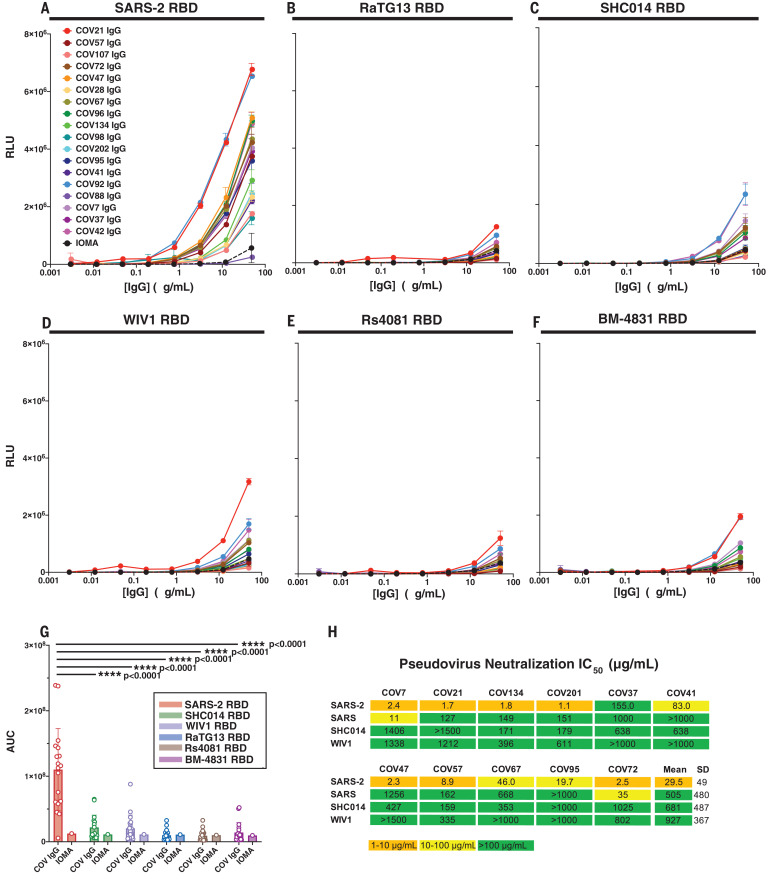
IgGs from convalescent COVID-19 plasma show little to no cross-reactive responses. (**A** to **F**) Plasma IgG (*18*, *24*) responses were evaluated by ELISA [data shown as binding curves with plasma names (*18*) listed] against RBDs from (A) SARS-2, (B) RaTG13, (C) SHC014, (D) WIV1, (E) Rs4081, and (F) BM-4831. Data points are plotted as means ± SD of duplicate measurements. IOMA, an anti–HIV-1 IgG (*50*), was used as a control. (**G**) ELISA results from (A) to (F), presented as AUC; each dot represents one plasma sample, with means ± SD shown as colored bars. Significant differences between groups linked by horizontal lines are indicated by asterisks and *P* values. (**H**) IC_50_ values for pseudotyped neutralization assays using IgGs from COV7, COV21, and COV72 plasmas (*18*) (evaluated at top concentrations of 1500 μg/ml) against the indicated strains. Mean = arithmetic mean IC_50_.

To compare antibodies elicited by RBD nanoparticle immunization to antibodies elicited by SARS-CoV-2 infection, we repeated ELISAs against the RBD panel using IgGs from COVID-19 plasma donors (*47*) (Fig. 4). Most of the convalescent plasmas showed detectable binding to SARS-2 RBD (Fig. 4A). However, binding to other sarbecovirus RBDs (RaTG13, SHC014, WIV1, Rs4081, and BM-4831) was significantly weaker than binding to SARS 2 RBD, with many human plasma IgGs showing no binding above background (Fig. 4, B to G). In addition, although convalescent plasma IgGs neutralized SARS-CoV-2 pseudoviruses, they showed weak or no neutralization of SARS, SHC014, or WIV1 pseudoviruses (Fig. 4H). These results are consistent with little to no cross-reactive recognition of RBDs from zoonotic coronavirus strains resulting from SARS-CoV-2 infection in humans.

Our results confirm that multimerization of RBDs on nanoparticles enhances immunogenicity relative to soluble antigen (*33*, *48*). We found that homotypic SARS-2 nanoparticle immunization produces IgG responses that bind zoonotic RBDs and neutralize heterologous coronaviruses after boosting. By contrast, soluble SARS-2 S immunization and natural infection with SARS-CoV-2 resulted in weak or no heterologous responses in plasmas. Co-display of SARS-2 RBD along with diverse RBDs on mosaic nanoparticles showed no disadvantages for eliciting neutralizing antibodies against SARS-CoV-2 relative to homotypic SARS-2 nanoparticles; therefore, mosaic nanoparticles may represent a candidate vaccine to protect against COVID-19. Furthermore, relative to homotypic SARS-2 RBD particles, the mosaic co-display strategy demonstrated advantages for eliciting neutralizing antibodies against zoonotic sarbecoviruses, thus potentially also providing protection against emerging coronaviruses with human spillover potential. Neutralization of matched and mismatched strains was observed after mosaic priming; hence, a single injection of a mosaic RBD nanoparticle might be sufficient in a vaccine. Because COVID-19 convalescent plasmas showed little to no recognition of coronavirus RBDs other than SARS-CoV-2, COVD-19–induced immunity in humans may not protect against another emergent coronavirus. However, the mosaic nanoparticles described here could be used as described or easily adapted so that they present RBDs from newly discovered zoonotic coronaviruses.

## Supplementary Materials

science.sciencemag.org/content/371/6530/735/suppl/DC1 Materials and Methods Figs. S1 to S5 References (*51*–*58*) MDAR Reproducibility Checklist

## Appendix

View/request a protocol for this paper from *Bio-protocol*.
